# Supplementary material for: Subnanometric Ru clusters with upshifted D band center improve performance for alkaline hydrogen evolution reaction
Source: Nat Commun. 2022 Jul 8;13:3958. doi: 10.1038/s41467-022-31660-2 (PMC9270335; doi:10.1038/s41467-022-31660-2)
Supplement: Supplementary file 1 — Supplementary Information [file 41467_2022_31660_MOESM1_ESM.pdf]

Subnanometric Ru clusters with Upshifted d Band Center Improve Performance for Alkaline Hydrogen Evolution Reaction

Q. Hu et al.,

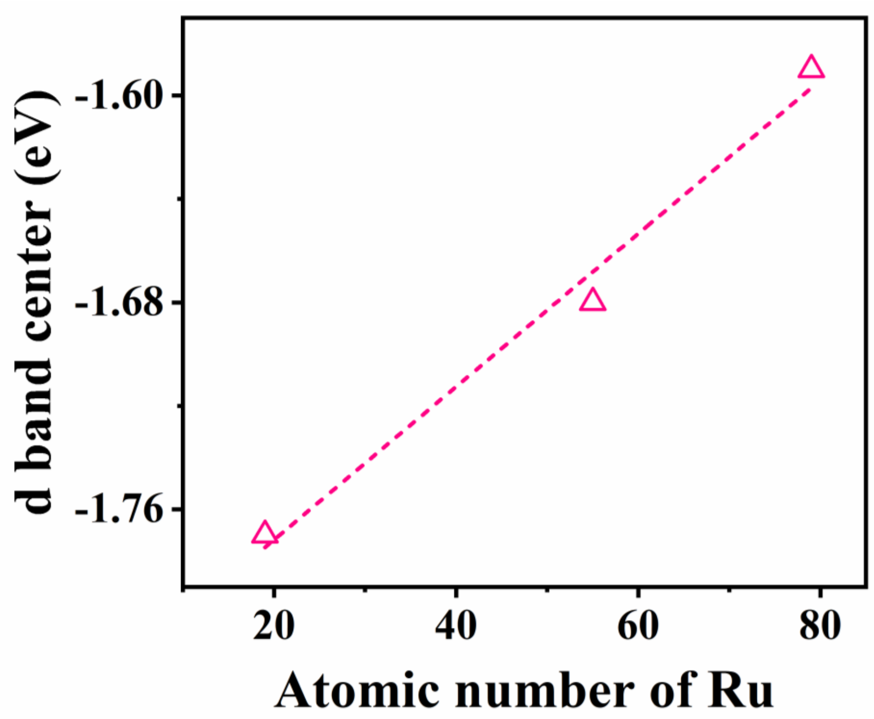

Supplementary Figure 1 Calculated d band center. Correlation between d band center of Ru cluster models and atomic number of Ru.

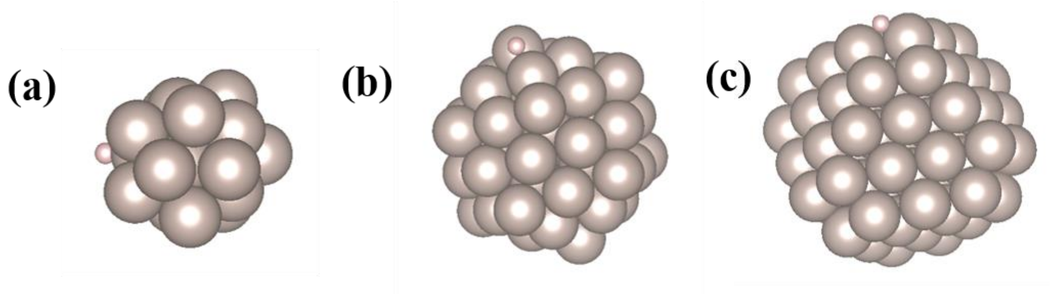

Supplementary Figure 2 Structure models for hydrogen adsorption. Optimized structures of hydrogen adsorbed on the (a) R_{19}, (b) Ru_{55}, and (c) Ru_{79} cluster models (19, 55, and 79 represent the atomic number of Ru). The silvery and light pink represent Ru and H atoms, respectively.

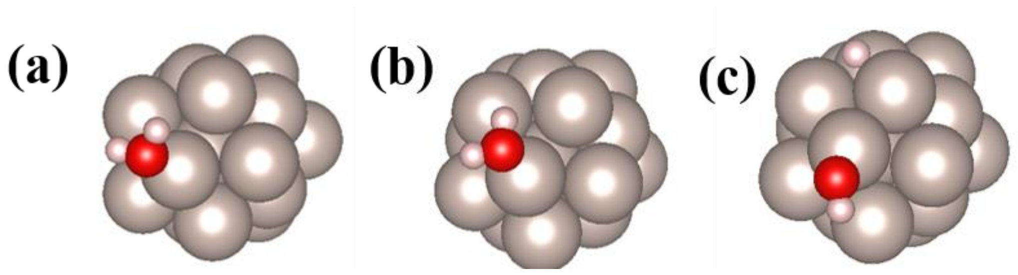

Supplementary Figure 3 Structural models of water dissociation on the Ru_{19} cluster. (a) initial state (IS), (b) transition state (TS), and (c) final state (FS). The silvery, red and light pink represent Ru, O, and H atoms, respectively.

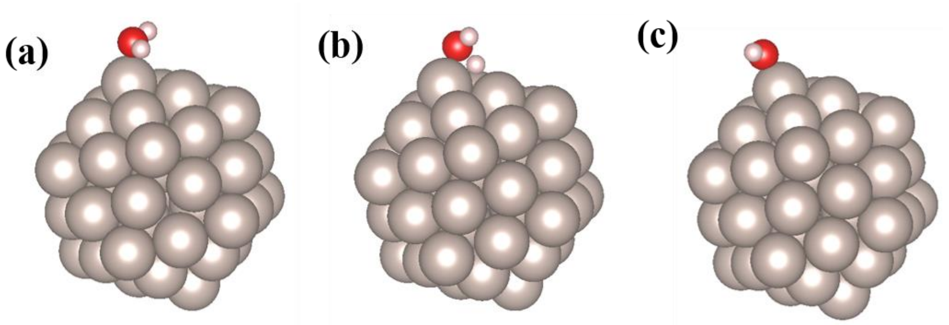

Supplementary Figure 4 Structural models of water dissociation on the Ru_{55} cluster. (a) initial state (IS), (b) transition state (TS), and (c) final state (FS). The silvery, red and light pink represent Ru, O, and H atoms, respectively.

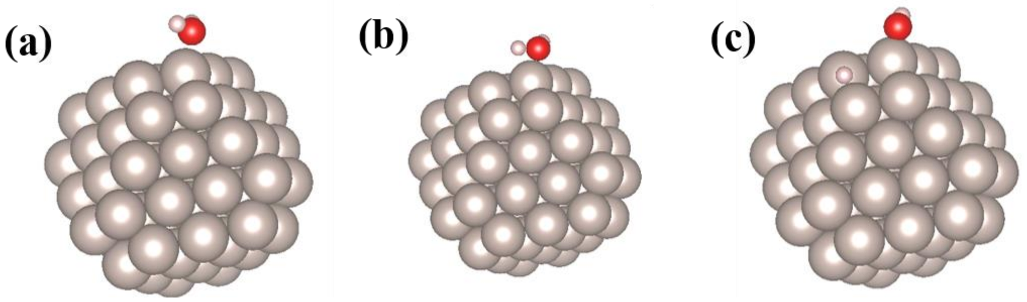

Supplementary Figure 5 Structural models of water dissociation on the Ru_{79} cluster. (a) initial state (IS), (b) transition state (TS), and (c) final state (FS). The silvery, red and light pink represent Ru, O, and H atoms, respectively.

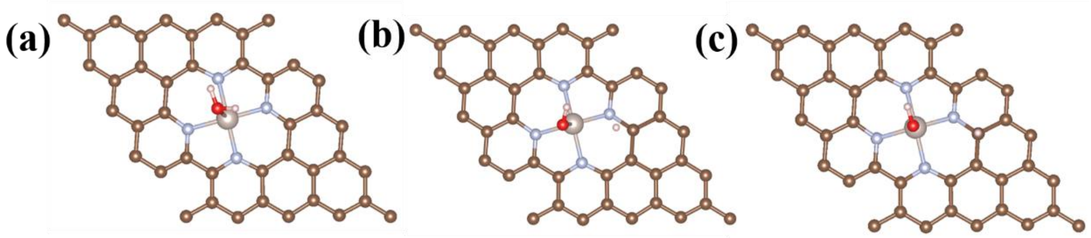

Supplementary Figure 6 Structural models of water dissociation on the RuN_4. (a) initial state (IS), (b) transition state (TS), and (c) final state (FS). The silvery, brown, red, light blue and light pink represent Ru, C, O, N, and H atoms, respectively.

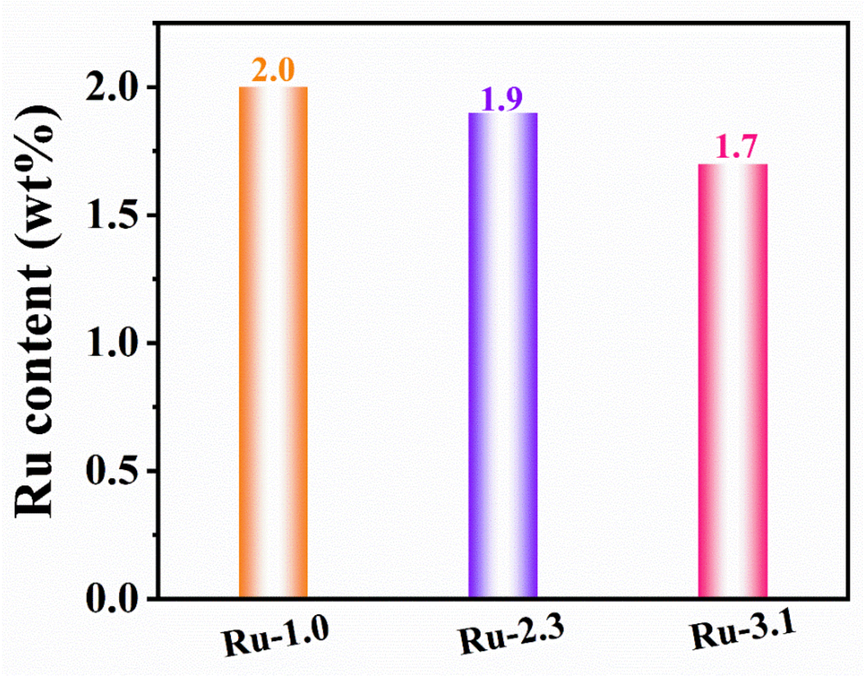

Supplementary Figure 7 Mass content of Ru. Comparing the Ru content on the Ru-1.0, Ru-2.3, and Ru-3.1.

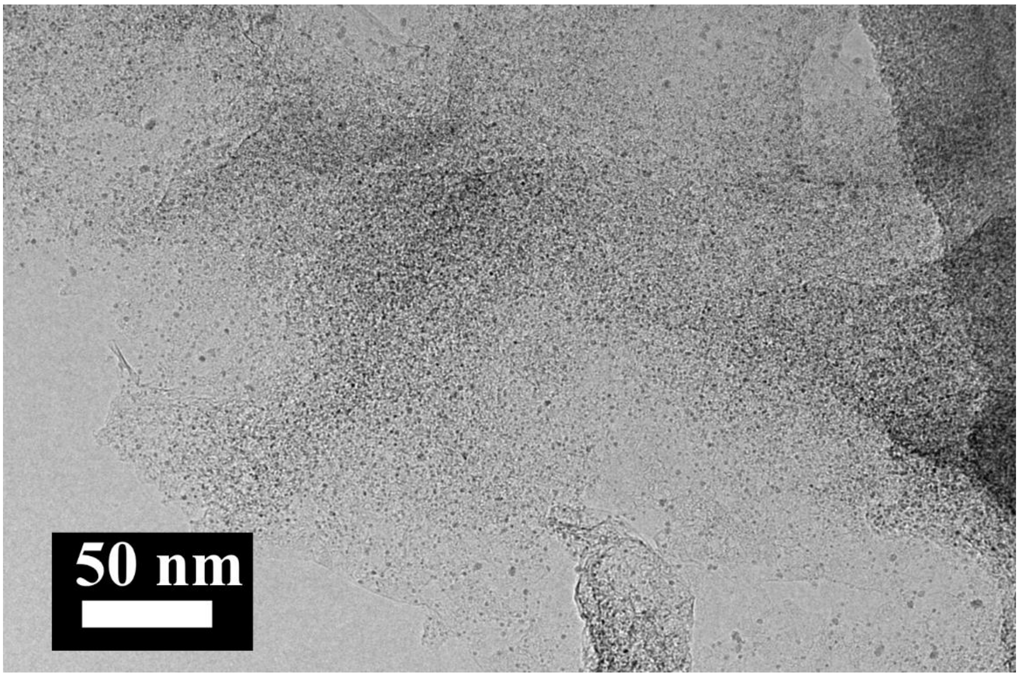

Supplementary Figure 8 Structural characterizations of Ru-1.0. TEM images of Ru-1.0

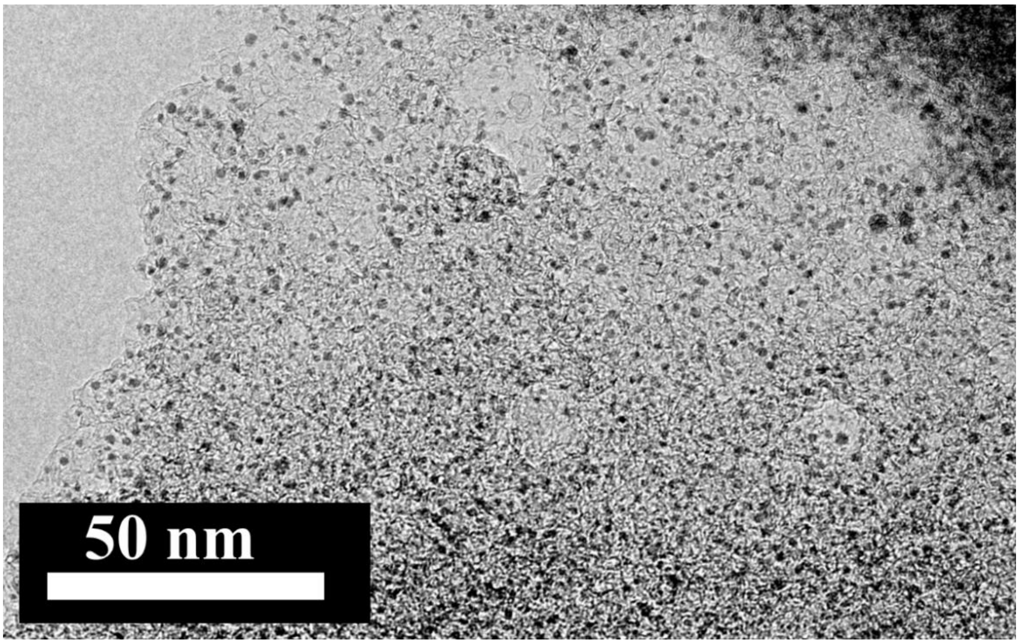

Supplementary Figure 9 Structural characterizations of Ru-2.3. TEM images of Ru-2.3.

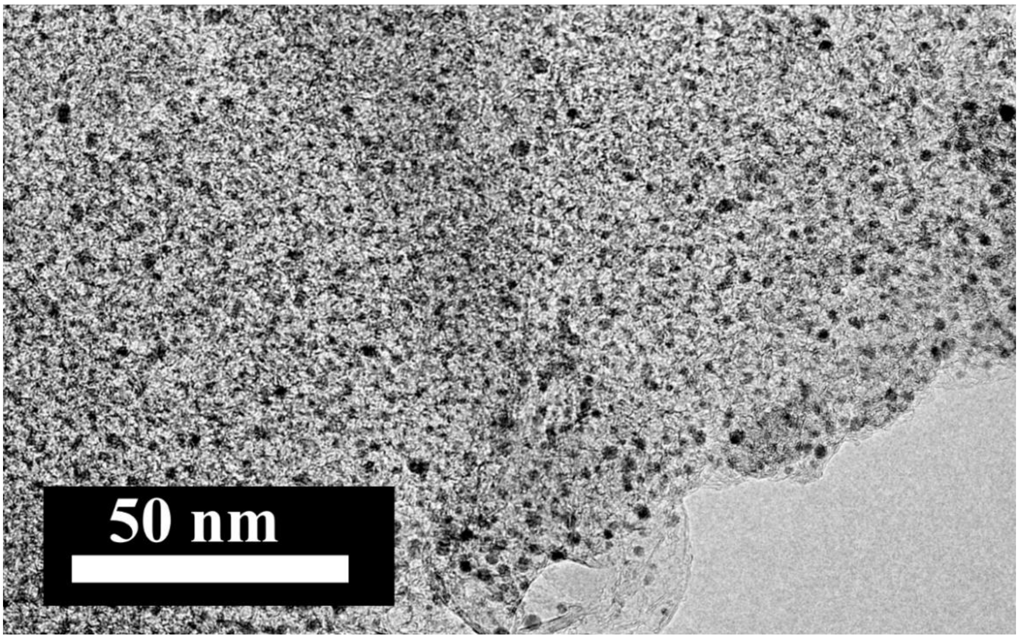

Supplementary Figure 10 Structural characterizations of Ru-3.1. TEM images of Ru-3.1.

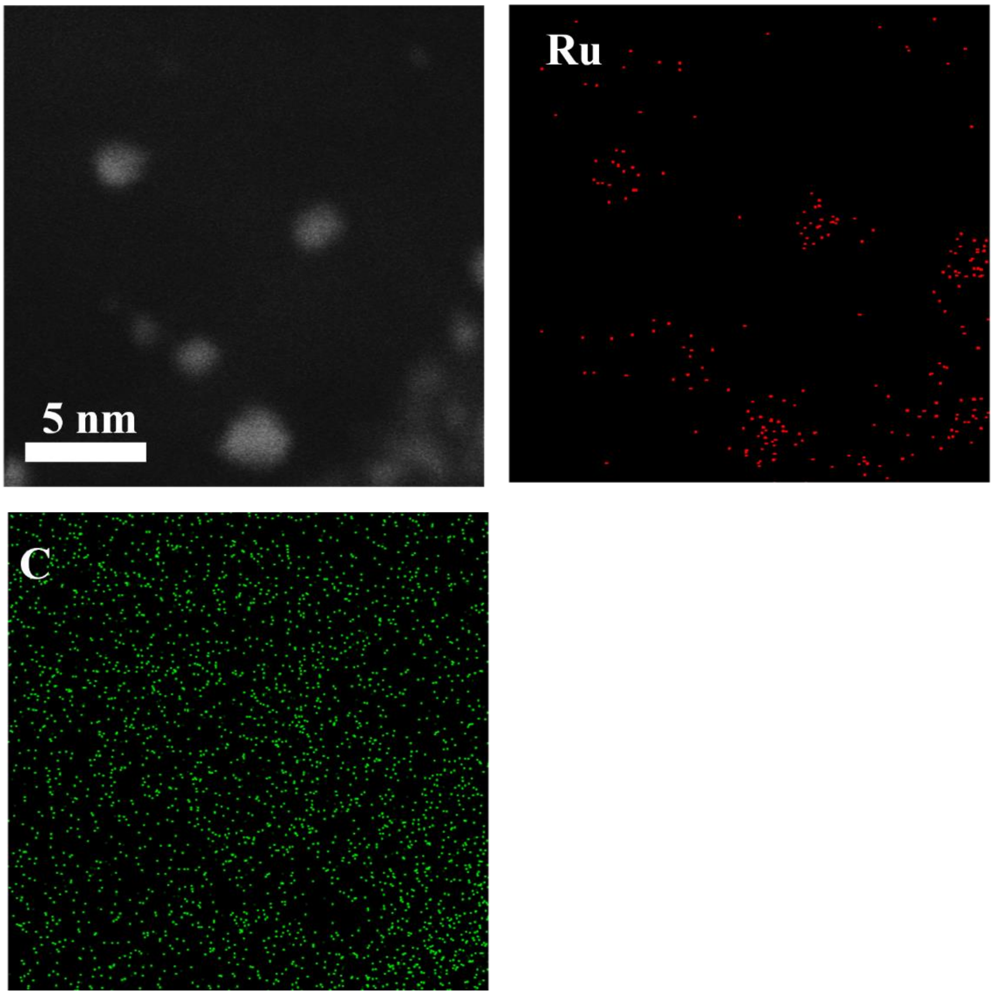

Supplementary Figure 11 Elemental mapping of Ru-1.0. HADDF-STEM image of Ru-1.0 and corresponding elemental mapping of Ru and C.

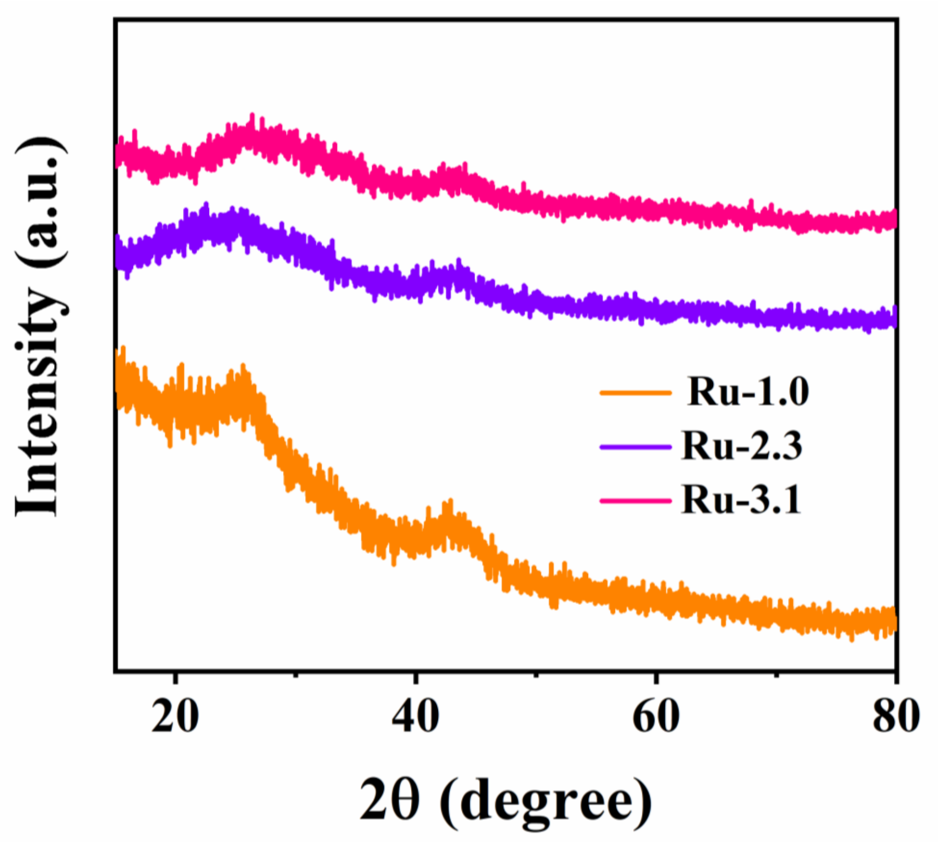

Supplementary Figure 12 Summary of XRD patterns. XRD patterns of Ru-1.0, Ru-2.3, and Ru-3.1.

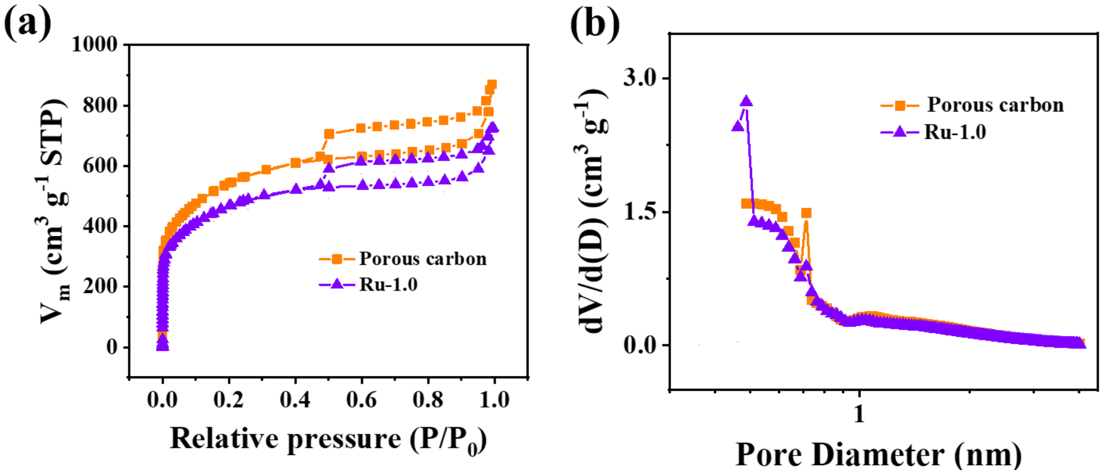

Supplementary Figure 13 BET surface area measurements. (a) N_2 adsorption-desorption isotherms of porous carbon and Ru-1.0, (b) corresponding pore distribution of above noted two samples based on a method of BJH.

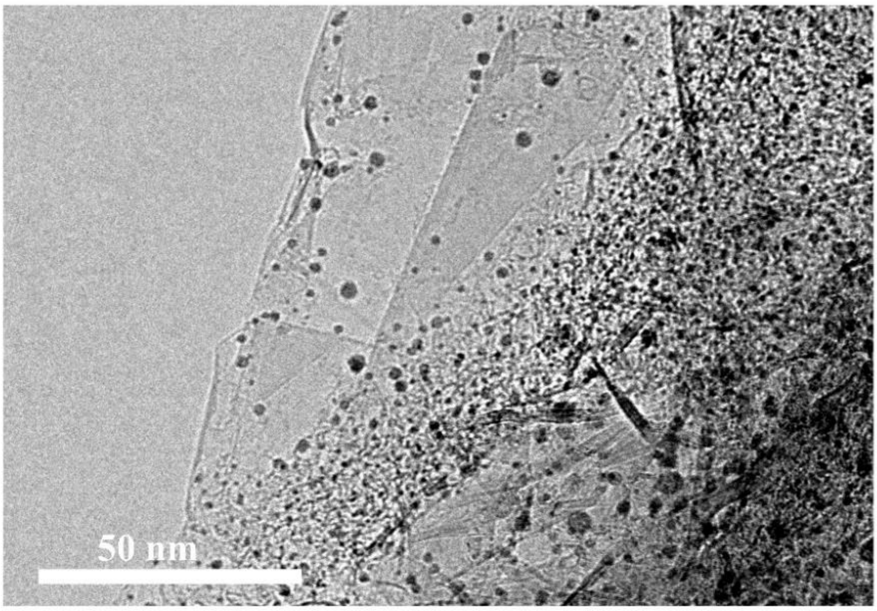

Supplementary Figure 14 Structural characterizations of control sample. TEM images of the sample prepared by using non-porous carbon as substrates.

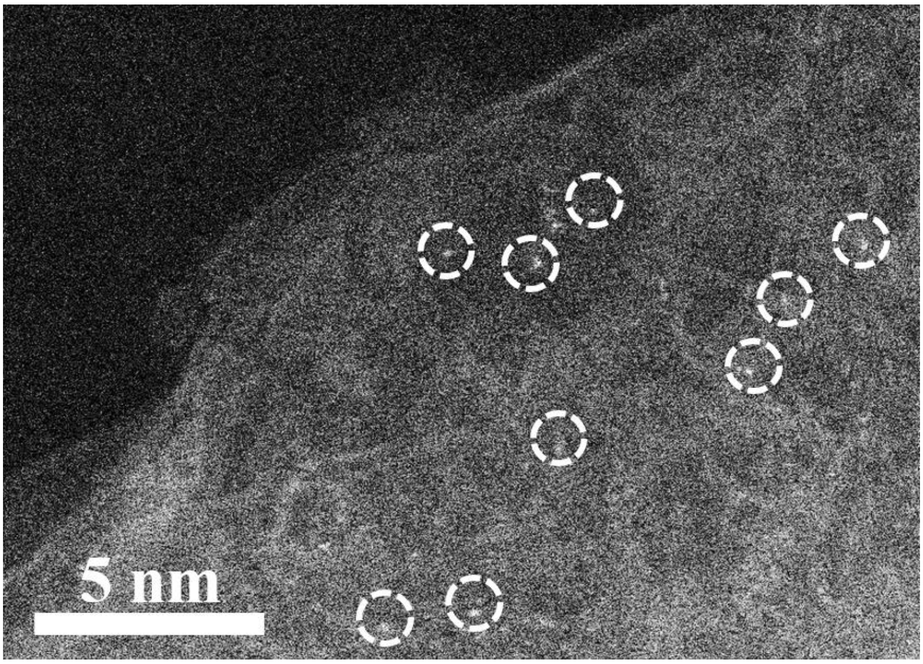

Supplementary Figure 15 Structural characterizations of single atomic Ru. HADDF-STEM images of Ru SAs.

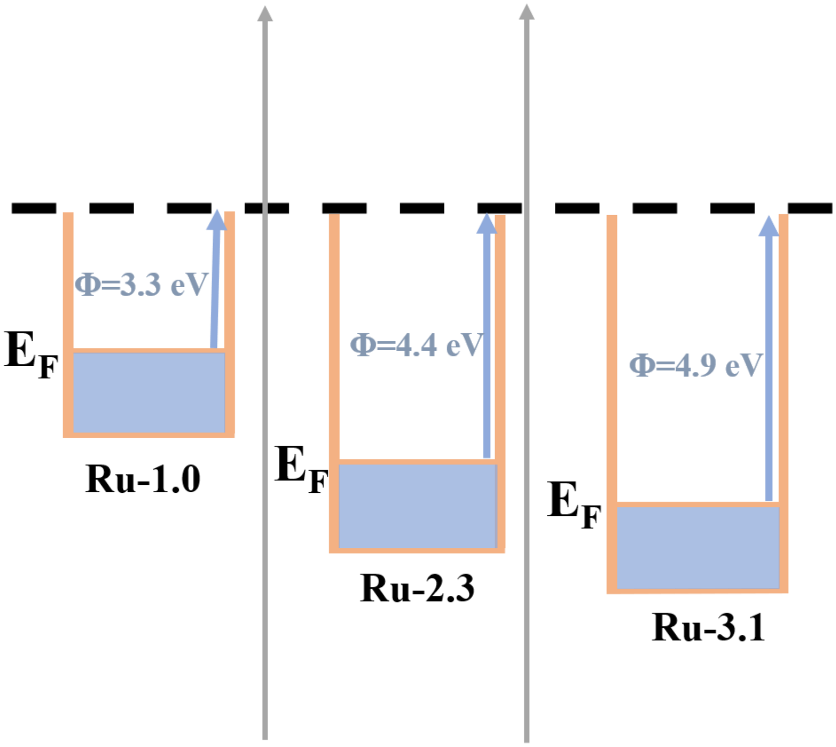

Supplementary Figure 16 Schematic illustrating the d band center change. The correlation between work functions and d band center of different Ru samples.

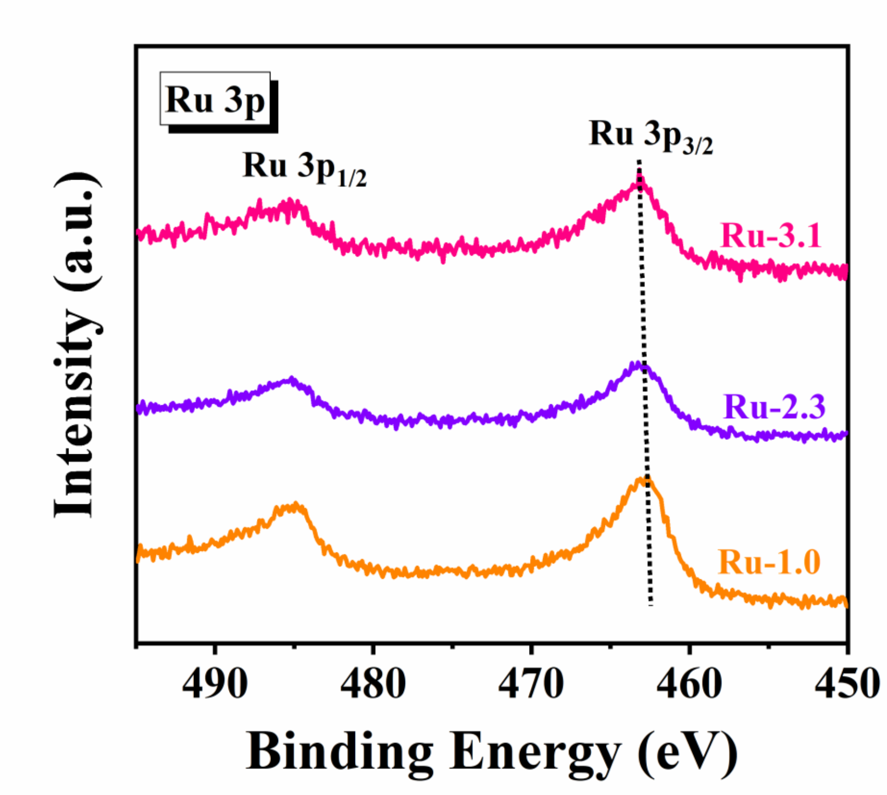

Supplementary Figure 17 Summary of XPS spectra. XPS spectra of Ru 3p for the Ru-1.0, Ru-2.3, and Ru-3.1.

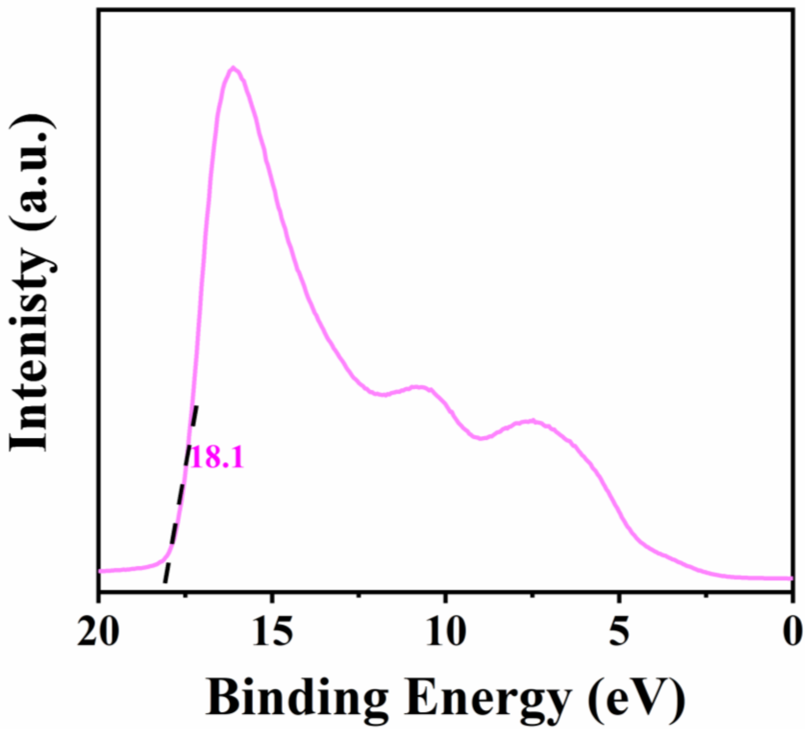

Supplementary Figure 18 Work function of Ru SAs. UPS spectra of Ru SAs.

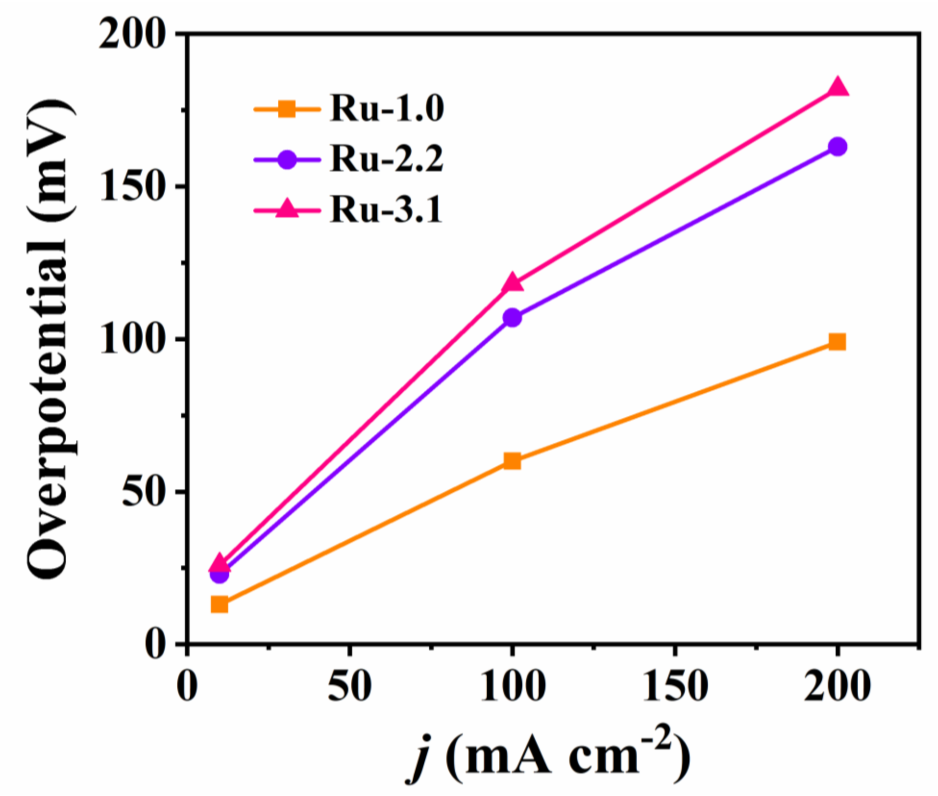

Supplementary Figure 19 Summary of overpotentials. Comparing overpotentials of Ru samples at different current densities.

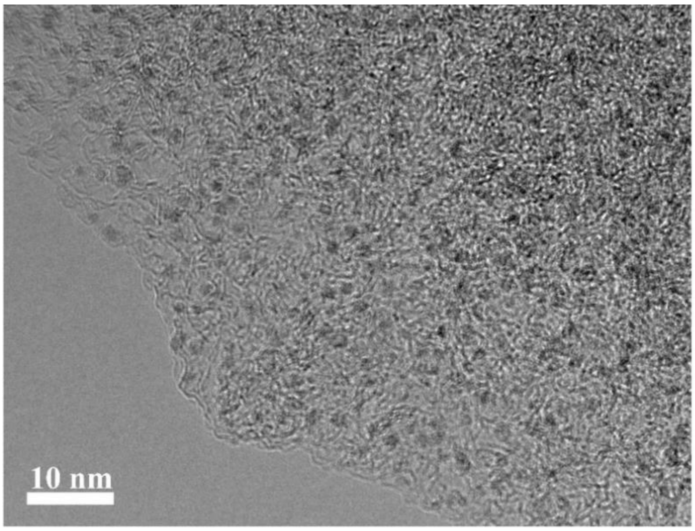

Supplementary Figure 20 Structural characterizations of Ru-1.0/N-free C. TEM images of Ru-1.0/N-free C.

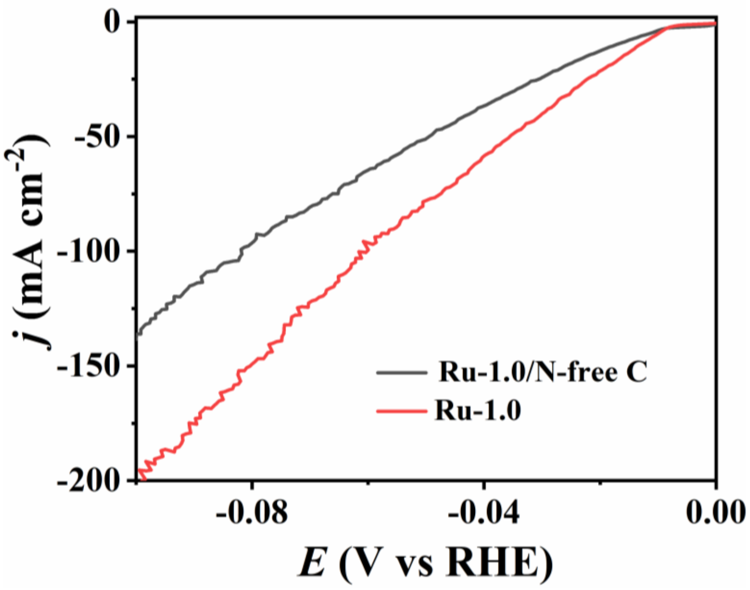

Supplementary Figure 21 LSV tests. LSV curves of Ru-1.0/N-free C and Ru-1.0 for the HER in 1 M KOH.

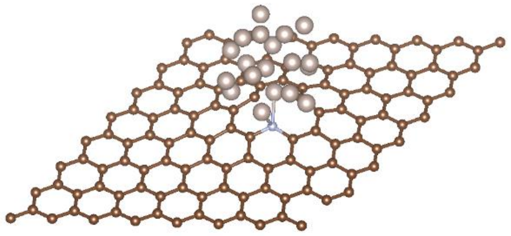

Supplementary Figure 22 Structural model of Ru_{19}/NC. The brown, ice blue, and silvery represent C, N, and Ru atoms.

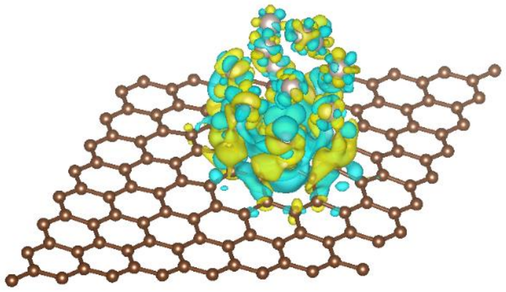

Supplementary Figure 23 Charge density difference diagrams. Three-dimensional charge density difference diagrams of Ru_{19}/NC.

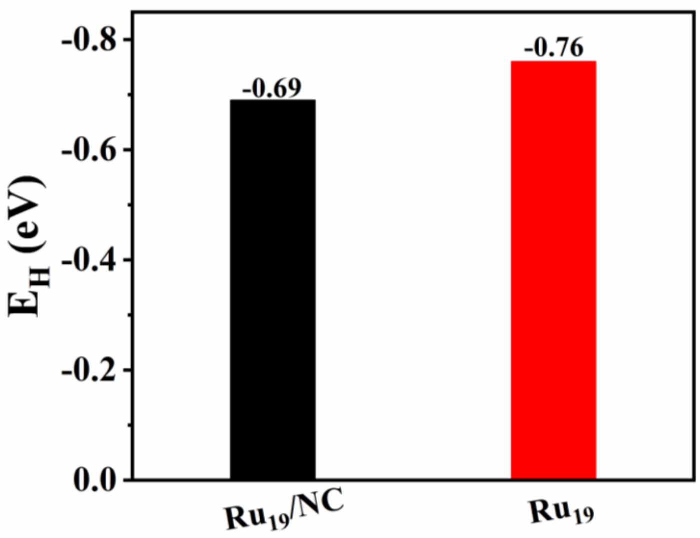

Supplementary Figure 24 Binding energies of hydrogen. Comparing binding energies of hydrogen on the structural model of Ru_{19}/NC and Ru_{19}.

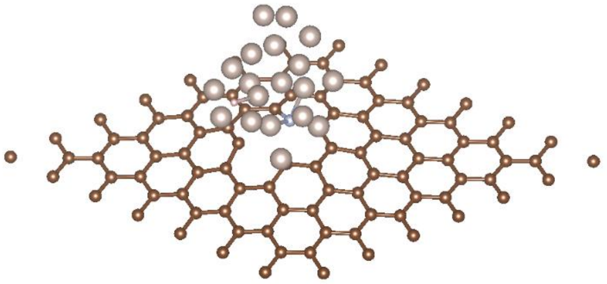

Supplementary Figure 25 Optimized structure of hydrogen adsorbed on the Ru_{19}/NC. The brown, ice blue, silvery, and light pink represent C, N, Ru, and H atoms, respectively.

$H_2O + e^- \longrightarrow H_{ads} + OH^-$ (Volmer) Tafel slope: 120 mV dec^{-1}

$H_{ads} + H_2O + e^- \longrightarrow H_2 + OH^-$ (Heyrovsky) Tafel slope: 40 mV dec^{-1}

$H_{ads} + H_{ads} \longrightarrow H_2$ (Tafel) Tafel slope: 30 mV dec^{-1}

Supplementary Figure 26 Elementary steps of alkaline HER. Tafel slope values corresponding to each elementary step.

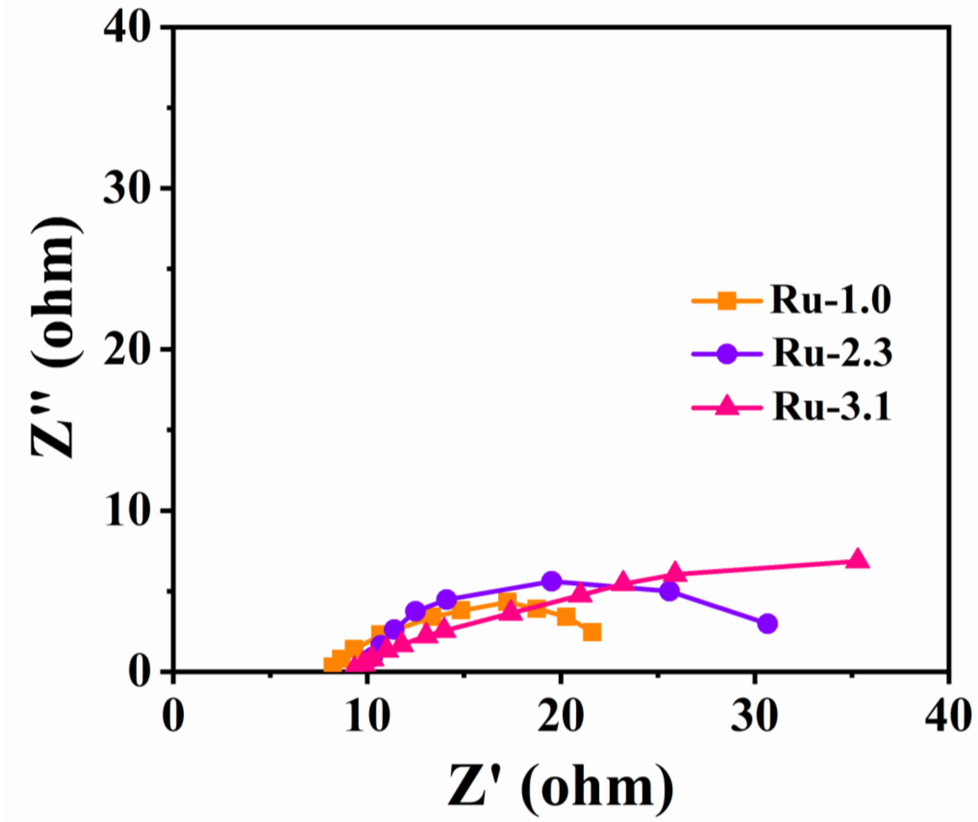

Supplementary Figure 27 Nyquist plots. Nyquist plots of various Ru samples at the overpotential of 20 mV.

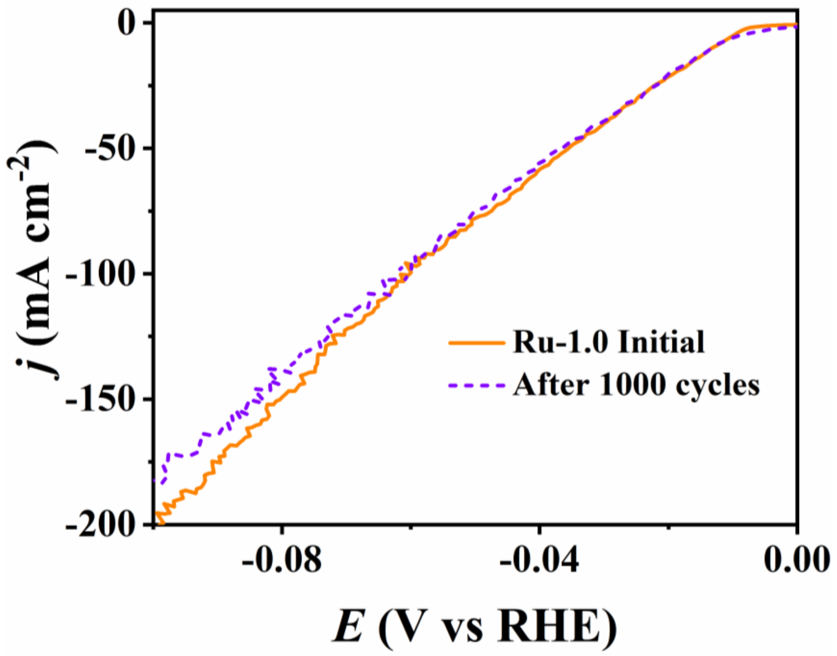

Supplementary Figure 28 Stability tests. LSV of Ru-1.0 before and after 1000 cycles of CV at the potential range from 0 to -0.4 V vs RHE.

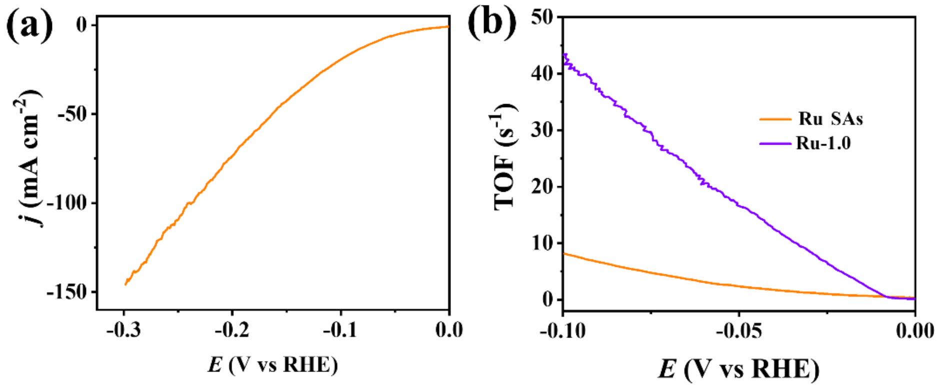

Supplementary Figure 29 Comparing HER performance of single atomic Ru and Ru clusters. (a) LSV of Ru SAs for HER in 1 M KOH. (b) Potential-dependent TOF curves of Ru SAs and Ru-1.0.

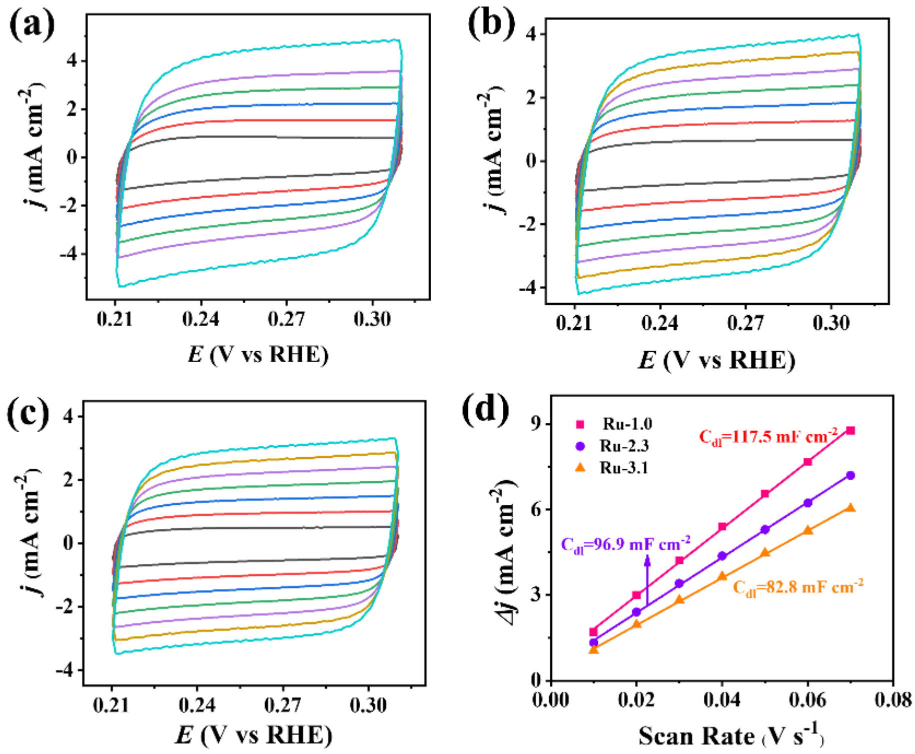

Supplementary Figure 30 Summary of ECSA tests. CV of (a) Ru-1.0, (b) Ru-2.3, and (c) Ru-3.1 at different scan rates over a potential range of 0.05-0.07 V vs RHE. (d) Profiles of capacitance Δj ($|j_{charge} - j_{discharge}|$) as a function of scan rates.

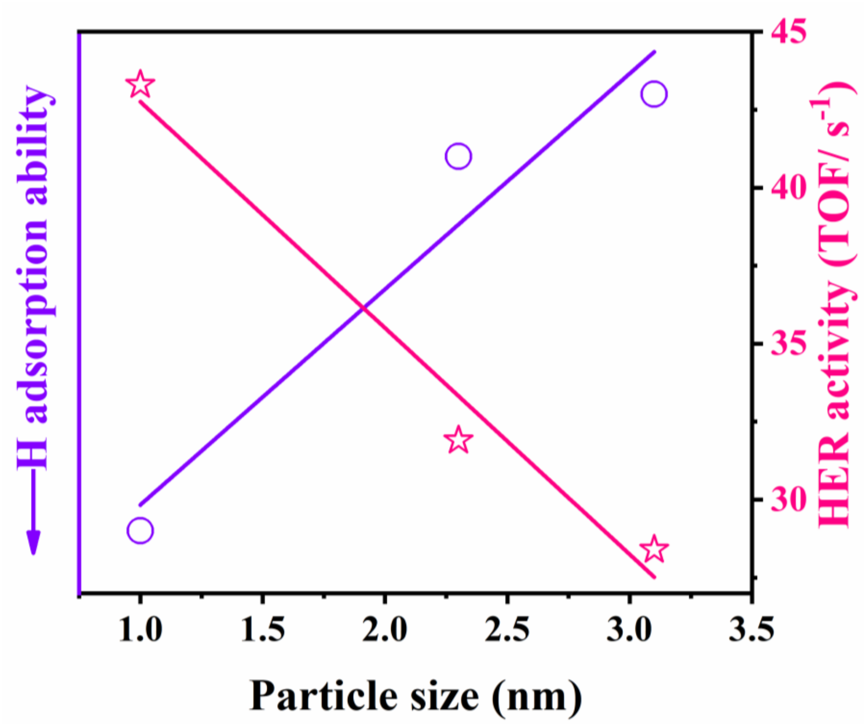

Supplementary Figure 31 Correcting the HER performance with H adsorption ability. Relationship of H adsorption ability, HER activity, and particle size of different Ru samples.

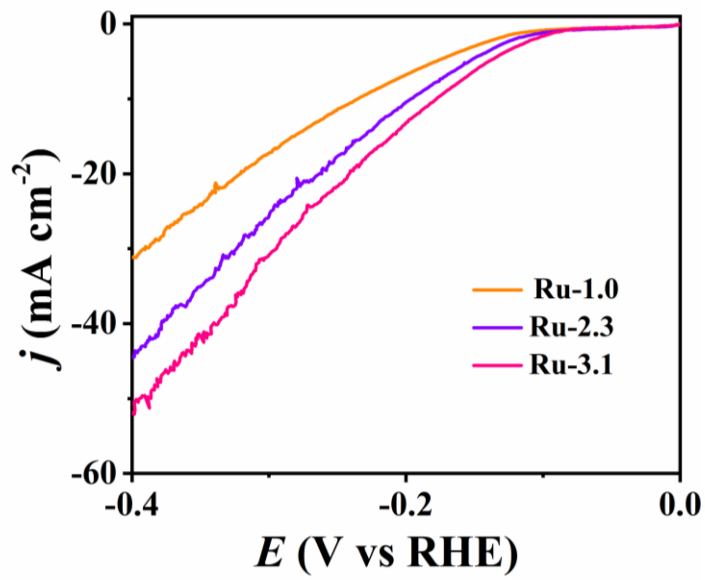

Supplementary Figure 32 HER performance in acid media. LSV curves of Ru-1.0, Ru-2.3, and Ru-3.1 for the HER in the electrolyte of 0.05 M H_2SO_4 solution.

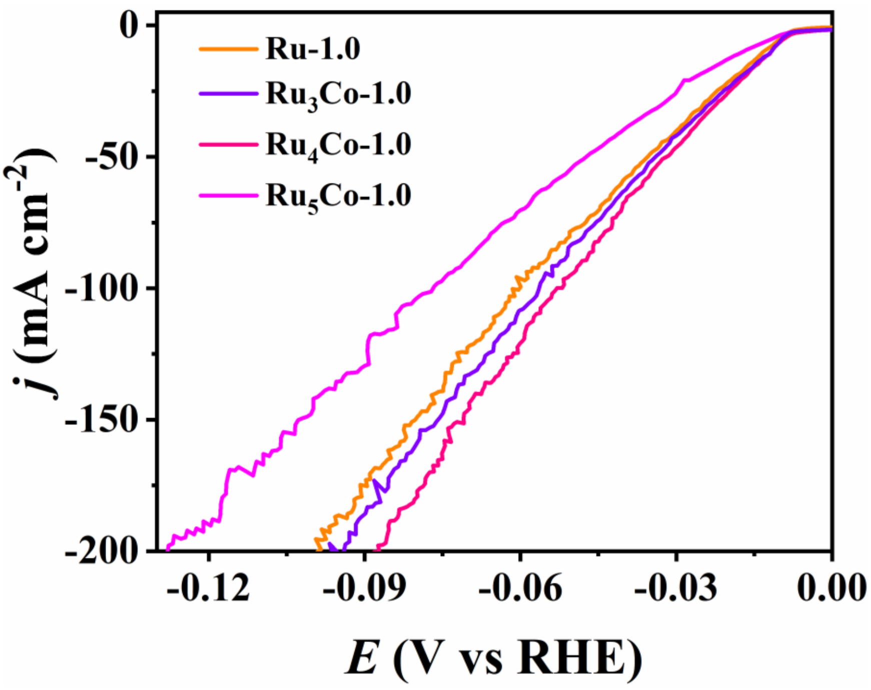

Supplementary Figure33 HER performance of samples with different content of Co. LSV of Ru-1.0, Ru_3Co-1.0, Ru_4Co-1.0, and Ru_5Co-1.0. (3, 4, and 5 represent the molar ration of Ru/Co)

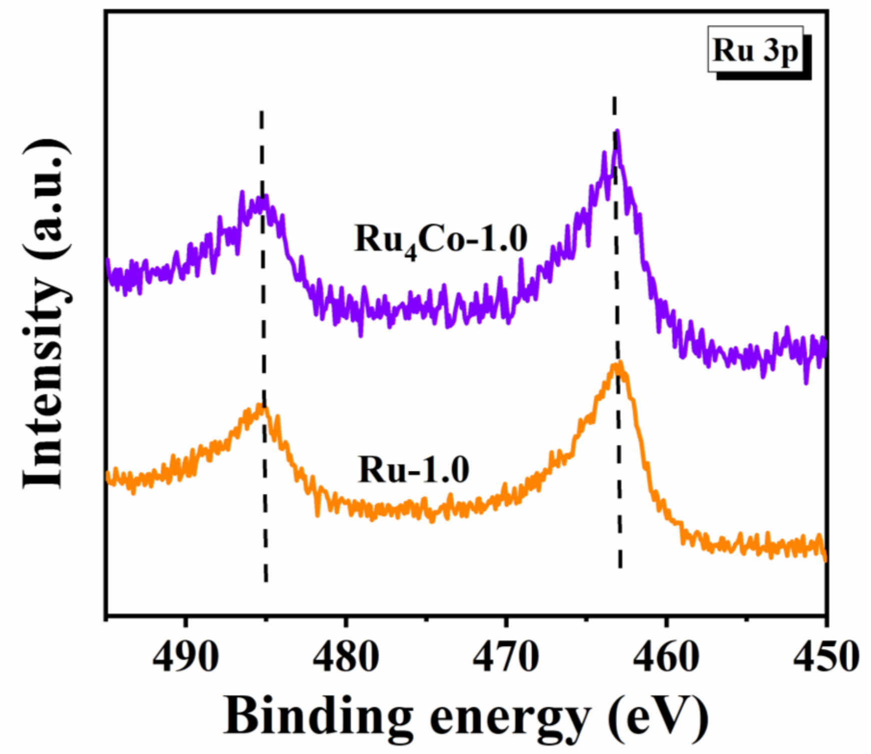

Supplementary Figure 34 XPS spectra. XPS spectra of Ru 3p for Ru-1.0 and Ru_4Co-1.0.

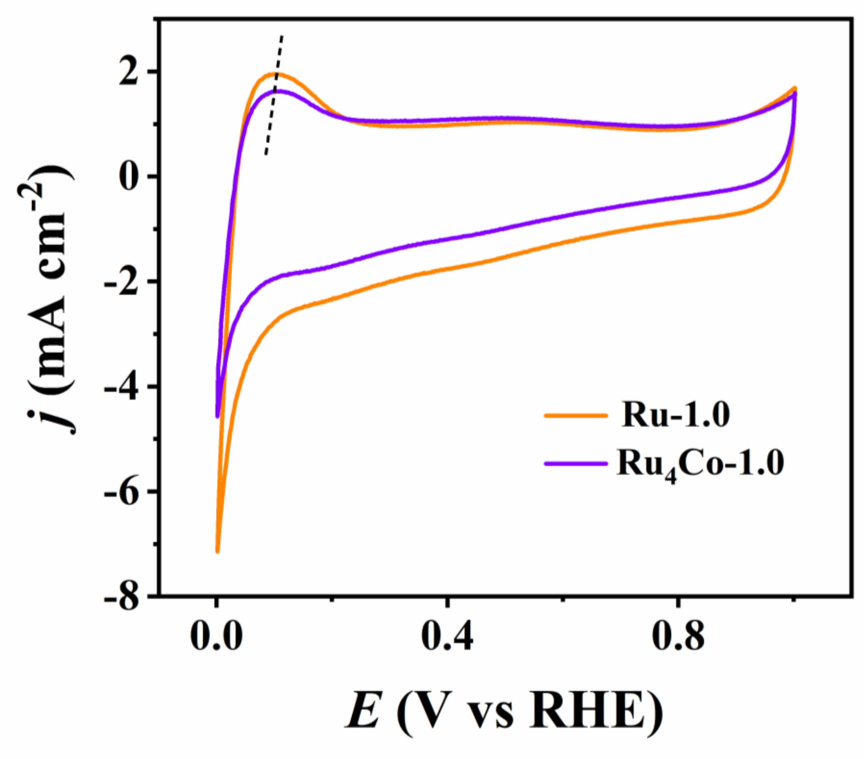

Supplementary Figure 35 Hydrogen binding energy. CV curves of Ru-1.0 and Ru_4Co-1.0 at the potential range of 0-1.0 V vs RHE.

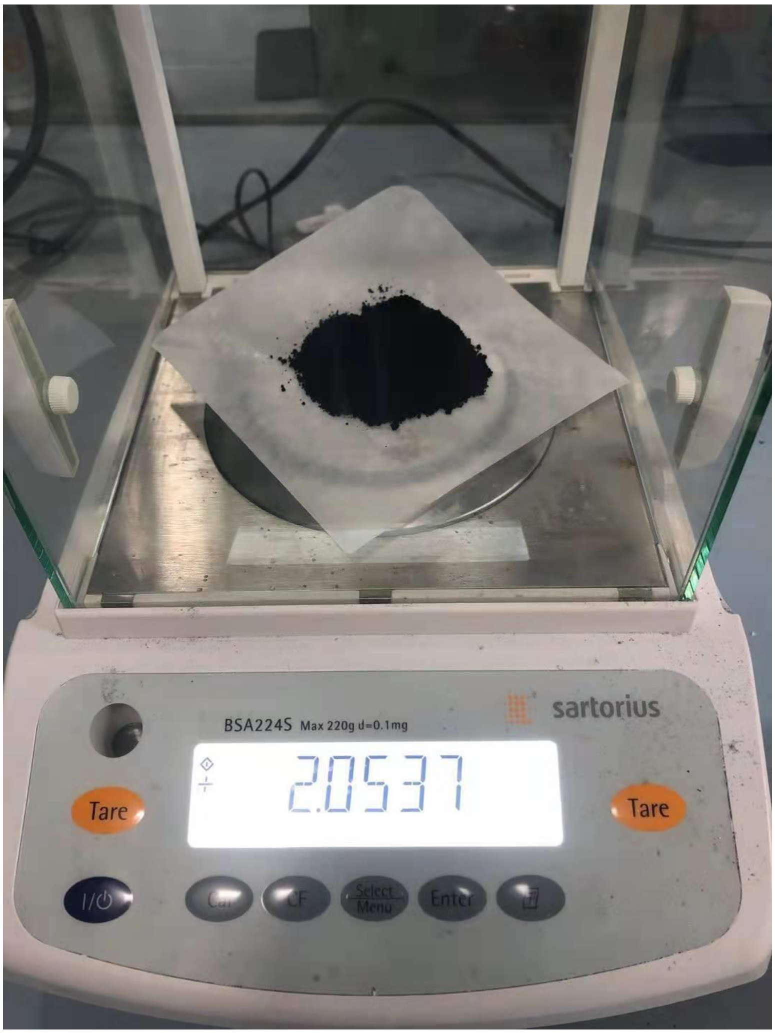

Supplementary Figure 36 Large-scale synthesis of Ru clusters. Photograph illustrating the large-scale synthesis of Ru-1.0 catalyst in one batch

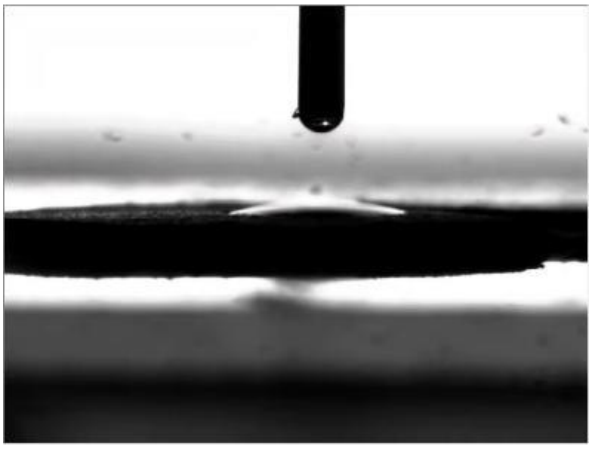

Supplementary Figure 37 Water contact tests. Water contact angle of Ru-1.0.

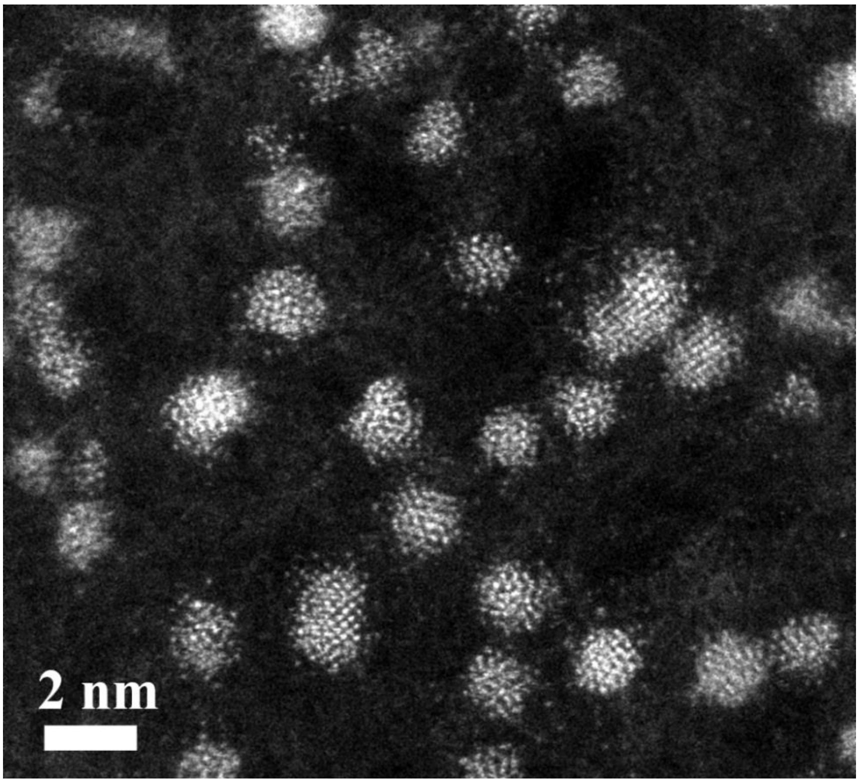

Supplementary Figure 38 Structural characterizations of Ru-1.0 after the stability test. HADDF-STEM images of Ru-1.0 after the stability at the current density of 1000 mA cm^{-2} for 1000 h

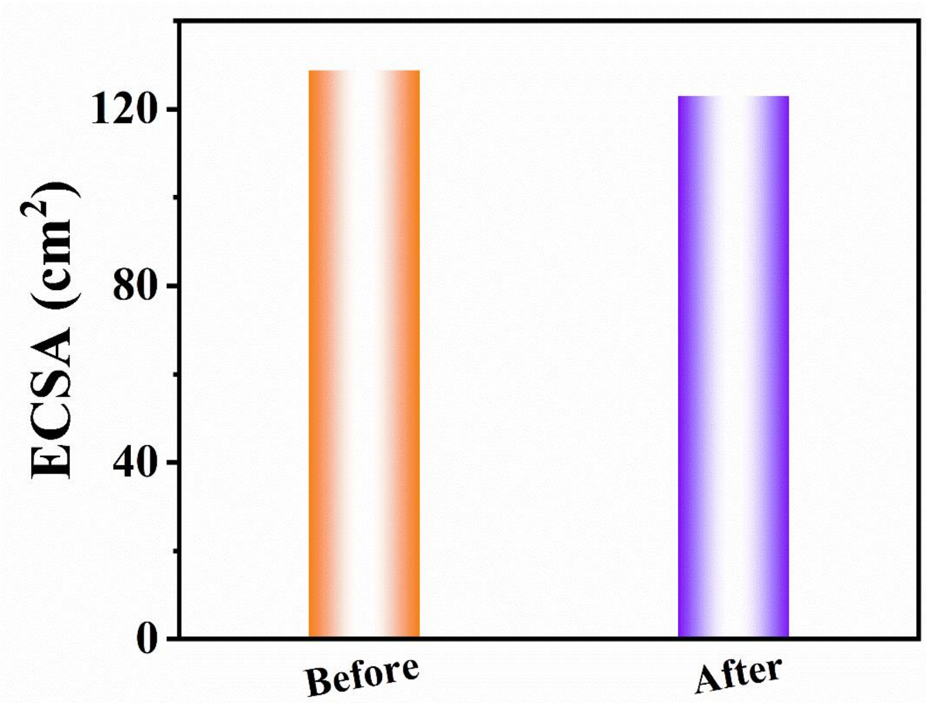

Supplementary Figure 39 ECSA values after the stability test. ECSA values of Ru-1.0 before and after the 100-h stability test determined by the CO-stripping experiment.

Supplementary Table 1 Comparing the HER activity of our Ru-1.0 with other reported electrocatalysts on basis of overpotential at 10 mA cm^{-2} and Tafel slope.

**Catalyst**	**Overpotential at 10 mA cm^{-2}**	**Tafel slope (mV dec^{-1})**	**Reference**
Ru-1.0	13	25	This work
Ru/C_3N_4/C	79	49	1
Ru@C_2N	17	38	2
RuP_2@NPC	52	69	3
RuCo@NC	28	31	4
Ru-Ni@Ni_2P-NRs	31	41	5
Au–Ru NWs	50	30.8	6
Ru@CN	32	53	7
ah-RuO_2@C	63	62	8
Ru@MWCNT	17	27	9
Ru_2P/WO_3@NPC	15	18	10
Ru-Mo_2C@CNT	15	26	11
Ru/OMSNNC	13	40	12
RuSA+NP/DC	19	36	13

Supplementary Table 2 Comparing the HER activity of our Ru-1.0 with other reported electrocatalysts on basis of TOF values.

**Catalyst**	**Overpotential (mV)**	**TOF (s^{-1})**	**Reference**
Ru-1.0	100	43.4	This work
Ru-1.0	10	1.2	This work
N,P-doped Mo_2C@carbon	10	3.71×10^{-3}	14
Ru@C_2N	25	0.75	2
$MoNi_4/MoO_{3-x}$	100	1.13	15
L-PR/C	20	3.64	16
Ru@NC	200	10.8	17
o-$CoSe_2$|P	200	14.95	18
Ru@Gnp	100	0.145	19
$CoP/Ni_5P_4/CoP$	100	1.22	20
P-Fe_3O_4/IF	150	0.242	21
2H $Nb_{1.35}S_2$	280	100	22
Ru@MWCNT	25	0.4	9
np-$Cu_{53}Ru_{47}$	100	1.139	23
Ru-Mo_2C@CNT	100	21.9	11

Supplementary Table 3 Comparing the HER activity of our Ru-1.0 with other reported electrocatalysts on basis of overpotentials at the current density of 1000 mA cm^{-2}.

**Catalysts**	**Overpotential (mV)**	**Reference**
Ru-1.0	196	This work
HC-MoS_2/Mo_2C	412	24
NiMoOx/NiMoS	236	25
Co(10.4)/Se-MoS_2-NF	382	26
Co-Mo_5N6	280	27
N_2P/NF	306	28
FeP/Ni_2P	293	29

Supplementary References

1 Zheng, Y. et. al. High Electrocatalytic Hydrogen Evolution Activity of an Anomalous Ruthenium Catalyst. *J. Am. Chem. Soc.* **138**, 16174-16181 (2016).

2 Mahmood, J. et. al. An efficient and pH-universal ruthenium-based catalyst for the hydrogen evolution reaction. *Nat. Nanotech.* **12**, 441–446 (2017).

3 Pu, Z. et. al. RuP_2-Based Catalysts with Platinum-like Activity and Higher Durability for the Hydrogen Evolution Reaction at All pH Values. *Angew. Chem. Int. Ed.* **56**, 11559–11564 (2017).

4 Su, J. et. al. Ruthenium-cobalt nanoalloys encapsulated in nitrogen-doped graphene as active electrocatalysts for producing hydrogen in alkaline media. *Nat. Commun.* **8**, 14969 (2017).

5 Liu, Y. et. al. Ru Modulation Effects in the Synthesis of Unique Rod-like Ni@Ni_2P–Ru Heterostructures and Their Remarkable Electrocatalytic Hydrogen Evolution Performance. *J. Am. Chem. Soc.* **140**, 2731-2734 (2018).

6 Liu, Q. et. al. Crystal phase-based epitaxial growth of hybrid noble metal nanostructures on 4H/fcc Au nanowires. *Nat. Chem.* **10**, 456–461 (2018).

7 Wang, J. et. al. Highly uniform Ru nanoparticles over N-doped carbon: pH and temperature-universal hydrogen release from water reduction. *Energy Environ. Sci.* **11**,

800-806 (2018).

8 Park, H.-S. et. al. RuO_2 nanocluster as a 4-in-1 electrocatalyst for hydrogen and oxygen electrochemistry. *Nano Energy*, **55**, 49-58 (2019).

9 Kweon, D.H. et. al. Ruthenium anchored on carbon nanotube electrocatalyst for hydrogen production with enhanced Faradaic efficiency. *Nat. Commun.* **11,** 1278 (2020).

10 Jiang, X. et. al. The Heterostructure of Ru_2P/WO_3/NPC Synergistically Promotes H_2O Dissociation for Improved Hydrogen Evolution. *Angew. Chem. Int. Ed.* **60**, 4110-4116 (2021).

11 Wu, X. et. al. Solvent-free microwave synthesis of ultra-small Ru-Mo_2C@CNT with strong metal-support interaction for industrial hydrogen evolution. *Nat. Commun*, **12**, 4018 (2021).

12 Wu, Y.-L. et. al. Ordered Macroporous Superstructure of Nitrogen-Doped Nanoporous Carbon Implanted with Ultrafine Ru Nanoclusters for Efficient pH-Universal Hydrogen Evolution Reaction. *Adv. Mater.* **33,** 2006965 (2021).

13 Zhang, L. et. al. Exploring the Dominant Role of Atomic- and Nano-Ruthenium as Active Sites for Hydrogen Evolution Reaction in Both Acidic and Alkaline Media. *Adv. Sci.* **8,** 2004516 (2021).

14 Chen, Y.Y. et. al. Pomegranate-like N,P-Doped Mo_2C@C Nanospheres as Highly Active Electrocatalysts for Alkaline Hydrogen Evolution. *ACS Nano* **10**, 8851–8860 (2016).

15 Chen, Y.Y. et. al. Self-Templated Fabrication of $MoNi_4/MoO_{3-x}$ Nanorod Arrays with Dual Active Components for Highly Efficient Hydrogen Evolution. *Adv. Mater.* **29**, 1703311 (2017).

16 Yu, J. et. al. Bigger is Surprisingly Better: Agglomerates of Larger RuP Nanoparticles Outperform Benchmark Pt Nanocatalysts for the Hydrogen Evolution Reaction. *Adv. Mater.* **30**, 1800047 (2018).

17 Wang, Z.L. et. al. Spatially confined assembly of monodisperse ruthenium nanoclusters in a hierarchically ordered carbon electrode for efficient hydrogen evolution. *Angew. Chem. Int. Ed.* **57,** 5848-5852 (2018).

18 Zheng, Y.-L. et. al. Doping-induced structural phase transition in cobalt diselenide

enables enhanced hydrogen evolution catalysis. *Nat. Commun.* **9**, 2533 (2018).
19 Li, F. et. al. Mechanochemically Assisted Synthesis of a Ru Catalyst for Hydrogen Evolution with Performance Superior to Pt in Both Acidic and Alkaline Media. *Adv. Mater.* **30**, 1803676 (2018).
20 Mishra, I.K. et. al. Hierarchical CoP/Ni_5P_4/CoP microsheet arrays as a robust pH-universal electrocatalyst for efficient hydrogen generation. *Energy Environ. Sci.* **11**, 2246-2252 (2018).
21 Zhang, J. et. al. Modulation of Inverse Spinel Fe_3O_4 by Phosphorus Doping as an Industrially Promising Electrocatalyst for Hydrogen Evolution. *Adv. Mater.* **31**, 1905107 (2019).
22 Yang, J. et. al. Ultrahigh-current-density niobium disulfide catalysts for hydrogen evolution. *Nat. Mater.* **18**, 1309-1314 (2019).
23 Wu, Q. et. al. Identifying Electrocatalytic Sites of the Nanoporous Copper–Ruthenium Alloy for Hydrogen Evolution Reaction in Alkaline Electrolyte. *ACS Energy Lett.* **5**, 192-199 (2020).
24 Zhang, C. et. al. High-throughput production of cheap mineral-based two-dimensional electrocatalysts for high-current-density hydrogen evolution. *Nat. Commun.* **11,** 3724 (2020).
25 Zhai, P. et. al. Engineering active sites on hierarchical transition bimetal oxides/sulfides heterostructure array enabling robust overall water splitting. *Nat. Commun.* **11**, 5462 (2020).
26 Zheng, Z. et. al. Boosting hydrogen evolution on MoS_2 via co-confining selenium in surface and cobalt in inner layer. *Nat. Commun.* 11, 3315 (2020).
27 Lin, F. et. al. Electrocatalytic Hydrogen Evolution of Ultrathin Co-Mo_5N_6 Heterojunction with Interfacial Electron Redistribution. *Adv. Energy Mater.* 10, 2002176 (2020).
28 Yu, X. et. al. "Superaerophobic" Nickel Phosphide Nanoarray Catalyst for Efficient Hydrogen Evolution at Ultrahigh Current Densities. *J. Am. Chem. Soc.* **141**, 7537-7543 (2019).

29 Yu, F. et. al. High-performance bifunctional porous non-noble metal phosphide catalyst for overall water splitting. *Nat. Commun.* **9**, 2551 (2018).
